# Efficacy and Safety of the All-Oral Schedule of Metronomic Vinorelbine and Capecitabine in Locally Advanced or Metastatic Breast Cancer Patients: The Phase I-II VICTOR-1 Study

**DOI:** 10.1155/2014/769790

**Published:** 2014-01-16

**Authors:** M. E. Cazzaniga, V. Torri, F. Villa, N. Giuntini, F. Riva, A. Zeppellini, D. Cortinovis, P. Bidoli

**Affiliations:** ^1^Oncology Department, AO S Gerardo, Via Pergolesi 33, 20900 Monza, Italy; ^2^Istituto di Ricerche Farmacologiche “Mario Negri” Via La Masa, 19 20156 Milano, Italy

## Abstract

*Background*. Vinorelbine (VRB) and capecitabine (CAPE) are demonstrated to be active in pretreated metastatic breast cancer patients. Different studies have demonstrated that the metronomic treatment is active with an acceptable toxicity profile. We designed a Phases I-II study to define the MTD of oral metronomic, VRB, and CAPE. *Patients and Methods*. Phase I: fixed dose of CAPE was 500 mg thrice a day, continuously. Level I of VRB was 20 mg/tot thrice a week for 3 weeks (1 cycle). Subsequent levels were 30 mg/tot and 40 mg/tot (Level III), respectively, if no Grades 3-4 toxicity were observed in the previous level. Phase II: further 32 patients received the MTD of VRB plus CAPE for a total of 187 cycles to confirm toxicity profile. *Results*. 12 patients were enrolled in Phase I and 22 in Phase II. Phase I: the MTD of VRB was 40 mg. Phase II: 187 cycles were delivered, observing 5.9% of Grades 3-4 toxicity. 31 patients are evaluable for efficacy, obtaining a clinical benefit rate of 58.1%. *Conclusion*. MTD of VRB with fixed dose of CAPE was 40 mg thrice a week and was the recommended dose for the ongoing Phase II multicenter study.

## 1. Background

Different studies in animal models have demonstrated that the combination of VRB and CAPE has a synergistic activity against breast cancer cells, due to their different mechanism of action [1]. These results have been subsequently confirmed in numerous studies conducted in metastatic breast cancer patients, heavily pretreated with taxanes and anthracyclines, in which VRB was administered as iv formulation [2].

Subsequent trials showed that there was a substantial equivalence between the iv and the oral formulations of VRB, even if this latter was characterized by a higher rate of haematological toxicity [3–5]. In all these studies, VRB and CAPE were administered on days 1 and 8, according to the approved standard schedule.

Metronomic chemotherapy refers to the frequent, even daily, administration of drugs at doses significantly lower than the maximum tolerated dose (MTD), with no prolonged drug-free breaks [6].

A recent study by Dellapasqua et al. [7] showed that the metronomic combination of cyclophosphamide and CAPE with bevacizumab was effective and minimally toxic in advanced breast cancer patients. This study fixed the dose of CAPE as 500 mg thrice a day, continuously.

Different studies have evaluated the possibility to administer VRB in a metronomic way [8, 9], trying to establish the MTD of the drug, both as single agent or as part of a multidrug regimen. These studies fixed the MTD of oral metronomic therapy with VRB at 40–60 mg/tot thrice a week.

No studies to our knowledge investigated the administration of an all-oral metronomic combination of the two drugs.

Aim of the present trial was to determine the MTD of metronomic VRB in combination with a fixed dose of CAPE in locally advanced or metastatic breast cancer patients. After defining the MTD, we conducted a Phase II study in order to verify the activity and the tolerability of the schedule.

## 2. Patients and Methods

This was a single-institution trial. Patients aged 18 years or more, with histological proven locally advanced (inoperable) or metastatic breast cancer with the following characteristics were eligible: pre- or postmenopausal, pretreated with anthracyclines and taxanes or not suitable for each of these drugs or both due to clinical conditions, HER2-negative or HER2-positive not suitable or no longer suitable to anti-HER2 agents due to cardiac impairment, measurable or evaluable disease, life-expectancy > 12 weeks, ECOG PS ≤ 2, adequate (bone) marrow, liver, and renal function (absolute neutrophil count > 1.5 × 10^9^/L, platelets > 100 × 10^9^/L, haemoglobin > 10 g/dL; total bilirubin within the normal institutional limits, AST/ALT < 2.5 × UNL, or < 5 × UNL in the case of liver involvement, creatinine within normal institutional limits, or creatinine clearance ≥ 50 mL/min, according to Cockroft-Gault formula), INR < 3 at the screening for patients taking warfarin, and absence of cerebral or leptomeningeal metastases. Written informed consent was required.

### 2.1. Study Treatment

The starting dose (Level I) of VRB was 20 mg thrice a week for 3 consecutive weeks (1 cycle). Each cohort was composed by three consecutive patients.

**Figure 1:**
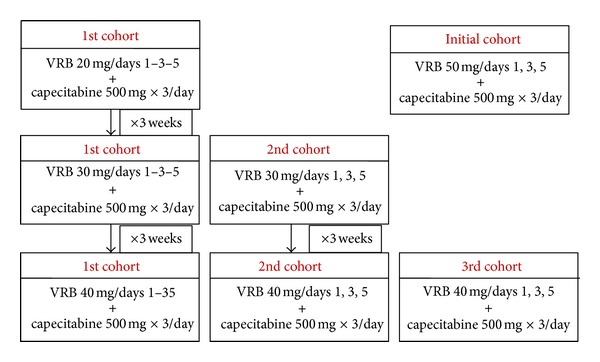
Study design.

Figure 1 describes the design of the study.

The first cohort started with Level I dose (20 mg), receiving the drug for 1 cycle. If no Grade 3-4 toxicity was observed during the 1st cycle, the first cohort escalated to Level II (30 mg) in the 2nd cycle, together with the initiation of the second cohort, which started with Level II directly in the 1st cycle. Both groups of patients received 1 cycle (3 weeks); if no Grade 3-4 toxicity was observed, they could increase the VRB dose to Level III (40 mg), together with the initiation of the third cohort, which directly began with 30 mg.

All patients who reached the level of 40 mg continued to be treated with the same dose up to disease progression, up to refusal to continue the study, and in any case up to 9 cycles of therapy (27 weeks overall). The analysis of toxicity was conducted considering the 1st cycle of each dose level. All toxicities were graduated according to the National Cancer Institute Common Terminology Criteria of Adverse Events (NCI-CTC, version 3).

The baseline evaluation included complete history and physical examination, assessment of performance status, CBC and differential, metabolic profile, coagulation studies, and serum pregnancy test in women with childbearing potential. Baseline staging was performed with total body CT scan, ultra sound for abdomen, standard chest X-ray, or clinical examination. Haematological toxicity was evaluated by CBC count every week and renal and liver function by biochemistry at the beginning of each subsequent cycle. Adverse events were collected each week.

### 2.2. Statistical Analysis

Patients were enrolled according to the aforementioned plan. Thus, for each MTD common toxicities (occurring in ≥30% of patients) would rarely be unobserved (*P* = 0.11), and very common toxicities (occurring in ≥50% of patients) would almost never be missed. Standard descriptive statistics were used for describing baseline characteristics and relevant safety and activity endpoints.

## 3. Results

### 3.1. Phase I

From October 2009 till April 2010, 12 patients have been enrolled. Median age was 72 years (49–81), 2 patients were metastatic *d'emblèe* at the time of enrolment, but they could not be treated with anthracyclines or taxanes due to cardiac impairment in the first case and refusal to have hair loss in the second one. ECOG performance status was 0-1 in 75% of the patients. median disease-free interval was 53 months (0–120). All patients were postmenopausal at the enrolment.

Table 1 reports the main characteristics of the patients enrolled in the study. Two patients were HER2+, but one has been already treated with trastuzumab, developing a cardiac heart failure which precluded any other therapy with anti-HER2 agents; the second could not receive any anti-HER2 treatment due to the presence of severe cardiomyopathy. In 3 patients HER2 status was not assessable due to little available histological material in 2 cases and paraffin block too old in another one. Labelling index (Ki67) was >10% in 9/12 patients (75%).

All patients but 3 had been treated with previous therapies, which were endocrine therapy in 2 patients, sequential chemotherapy and endocrine therapy in 6, and chemotherapy plus trastuzumab in 1 case.

Nine patients presented 2 or more metastatic sites; the remaining patients had just one organ involved.

Seven out of 12 (58.3%) patients received anthracyclines, of whom 6 were treated with anthracycline plus paclitaxel combination and 7 received taxanes, alone (1 patient) or in combination with anthracyclines (6 patients). Four patients received VRB plus CAPE as first-line treatment of their metastatic disease because of their comorbidities, which precluded the use of other drugs.

A total of 25 cycles were administered and analysed for toxicity. Three cycles have been conducted at Level I (20 mg), 6 cycles at Level II, and 16 cycles at Level III.

Details concerning adverse events per cycle are described below and resumed in Table 2.

#### 3.1.1. Level I (VRB = 20 mg)

All 3 patients of the first cohort completed the planned cycle of 3 weeks; no delay or dose reduction was required. In Level I we observed 4 adverse events per cycle: 2 constipation G2, 1 nausea G1, and 1 dyspnoea G1. No haematological event was observed.

No G3-4 toxicity has been observed.

#### 3.1.2. Level II (VRB = 30 mg)

Six patients were treated at Level II for a total of 6 cycles, without any delay, dose reduction, or suspension. We observed a total of 15 adverse events per cycle, of which 11 were classified G1 (73.3%); abdominal pain was reported in 3 cases, nausea in 4, and gastric pain, stomatitis, and asthenia in 1. Grade 2 adverse events were reported in 4 cases: nausea 2 and constipation 2. No haematological toxicity was observed.

#### 3.1.3. Level III (VRB = 40 mg)

Eight patients were treated with VRB 40 mg for a total of 16 cycles. One patient of the second cohort did not complete the planned treatment because of death due to liver metastases at the end of Level II dose. We observed a total of 46 adverse events, Grade 1 in 38 cases (82.6%); the majority of them concerned the gastrointestinal area (33 events, 86.8%).

Regarding haematological toxicity, we observed 2 events of leukopenia and 2 cases of anaemia, in all cases Grade 1.

The MTD dose of metronomic VRB in combination with fixed doses of CAPE was established at 40 mg days 1, 3, and 5 of each week.

### 3.2. Phase II

In order to confirm the toxicity profile observed in the dose-finding part of the study, we further treated 22 patients with VRB 40 mg and CAPE 500 mg thrice a day, for a total of 187 cycles. Patients' characteristics are detailed in Table 1. We observed 90 Grade 1 events (48.1%), 29 Grade 2 (15.5%), 7 Grade 3 (0.03%), and 4 Grade 4 events (0.02%) per cycle. Among Grade 3 events, 2 neutropenia (0.01%) and 1 thrombocytopenia (0.005%) events were reported. Grade 3 neutropenia was associated to Grade 1 leukopenia, which required 1-week delay in chemotherapy administration; the event spontaneously recovered and the patients continued her therapy at the same dose. One case of hand-foot syndrome was described, which required the reduction of CAPE to 500 mg bid. Grade 3 neurological toxicity occurred in 1 patient and VRB was reduced to 20 mg/tot. Among Grade 4 events, 2 neutropenia events of which 1 of febrile neutropenia, and 2 leukopenia events occurred. Febrile neutropenia was complicated by the presence of erysipelas and required hospitalization and antibiotic treatment with amoxicillin and clindamycin, together with G-CSF administration for 5 days. In this case, chemotherapy was suspended.

Efficacy was assessed according to RECIST 1.0 criteria. Tumour restaging was performed every 3 cycles (9 weeks). At the 3rd evaluation (27 weeks of treatment), patients who did not progress accounted for the clinical benefit. Thirty-one patients were evaluable for efficacy: 3 patients in the Phase I part did not reach the timing of tumour evaluation due to rapid progression in 2 patients and development of febrile neutropenia which determined definitive suspension of the treatment in the reaming one.

Clinical benefit defined as CR + PR + SD ≥ 24 weeks was observed in 18/31 patients (58.1%), (Table 3).

## 4. Discussion

The present study was designed to establish the MTD of oral metronomic VRB in association with fixed metronomic doses of CAPE. To our knowledge, this is the first study which investigates the combination of a full oral, metronomic schedule of VRB and CAPE.

Assuming that VRB and CAPE have different toxicity profiles and no enhanced toxicity should be expected from the combination, we initially designed a study to evaluate the clinical activity of the combination. VRB was planned to be administered at the dose of 50 mg every other day, which was the recommended dose coming from the studies of Briasoulis et al. [8], whereas CAPE dose was 500 mg, tid, which was the dose used by Dellapasqua et al. [7]. Despite the results of the above-mentioned study, we excluded this dose from the combination with CAPE 500 mg tid continuously, because of the occurrence of one event of G4 neutropenia and one event of G3 neurological toxicity, clearly related to VRB instead of CAPE, in 2 out of the first 3 patients. Considering that these toxicities were strongly related to the administration of VRB instead of CAPE, we reviewed our initial intention by designing the present intrapatient, dose-finding study, with no modifications of CAPE dose and assuming 50 mg thrice a week of VRB as the limit dose.

In the study by Briasoulis et al., the dose of VRB 50 mg determined just 3 adverse events, all of them being G1-2 (anaemia 1 and nonhaematological 2).

The patient who developed G4 neutropenia has received 6 cycles of epirubicin and paclitaxel as first-line treatment for the metastatic disease and her blood reserve could have been compromised by that treatment. The patient who developed 3 neurological pain events was in excellent general condition at the beginning of the protocol, without any preclinical neurological condition.

During the 25 cycles delivered in the dose-finding part of the study, no Grade 3-4 adverse events occurred in our patients. A total of 51 Grade 1 events were described, most of them regarding the gastrointestinal area (45 events, 88.2%) and 14 Grade 2, 8 of them were concerning the same area.

Different studies [5, 10–14] have evaluated the efficacy and the safety of oral VRB in combination with CAPE; most of them used VRB at doses ranging from 60 to 80 mg/mq, administered on days 1 and 8 every 21 days, as the classical regimen. In all those studies, CAPE was administered at a dose ranging from 800 to 1250 mg/mq bid, from day 1 to day 14, every 21 days. None of them used the metronomic schedule.

Five trials [5, 10–12] studied the combination of VRB 60 mg/mq (days 1–8) and CAPE 2000 mg/mq/day, days 1–14, every 3 weeks.

In the study by Lorusso et al. [10], 38 advanced breast cancer patients received a total of 228 courses. The authors observed Grade 2-3 neutropenia in 18.9% and Grade 4 in 2.7%, thrombocytopenia Grade 3 in 2.7%, and nausea/vomiting Grade 3 in 2.7% of the patients, concluding that that regimen was safe and easy to administer in an outpatient setting. No data concerning the duration of the treatment was reported.

Tubiana-Mathieu et al. [5] reported a 49% of Grade 3-4 neutropenia treating 54 patients for a median number of 7 cycles (range 1–58); in addiction, 2 patients experienced febrile neutropenia (3.8%) and 3 additional patients had documented infection associated with Grade 3-4 neutropenia, one of them producing a fatal septicaemia.

A similar but larger Phase II study was conducted by Finek et al. [12] reporting a 0.8% incidence of Grade 4 neutropenia in approximately 115 patients.

Nolè et al. [11] treated 44 patients with metastatic breast cancer with the same dose of oral VRB 60–80 mg/mq and escalating doses of CAPE from 1650 to 2500 mg/mq/day, days 1–4 every 3-4 weeks. Neutropenia was the main dose-limiting toxicity of the combination; it was reported in 40 patients (90.0%), with Grade 3 in 14 patients (31.8%) and 6.2% of the cycles and Grade 4 in 12 patients (27.3%) and 4.3% of the cycles. The authors also reported a frequent gastrointestinal toxicity, even if the incidence of Grade 3 was low, with no episode of Grade 4. Nevertheless, cycle delay occurred in 61.4% of the patients and 26.12% of the cycles. Day 1 oral VRB administration was cancelled in 38.6% of the patients and 7.7% of the cycles and dose reduction occurred in 22 patients (50%) and 7.4% of the cycles.

In the study by Jones et al. [14], forty patients received a median number of 4 cycles (range 1–31); main toxicity was haematological, with 7.9% of Grade 3 and 3.5% of Grade 4 neutropenia per cycles. Febrile neutropenia occurred in 0.4% of the cycles.

Comparing to the standard administration of the combination of VRB and CAPE, we observed a significantly lower incidence of Grade 3-4 events; this could be explained by the metronomic schedule of administration, which, by definition, consists of low, repeated doses of the same drug without rest period. This kind of administration warrants the delivery of similar, if not superior, dose intensity than the 1–8 schedule of VRB: if we consider the definition of a cycle as a 3-week period and a median body surface area (BSA) of 1.6 for women, the standard schedule provides the administration of approximately 192 mg/tot/cycle, in comparison to 360 mg/tot/cycle of the metronomic regimen. The delivered dose of CAPE in the standard schedule would be 44800 mg/tot, in comparison to 31500 mg/tot of the metronomic schedule. One could argue that the dose of CAPE delivered in the metronomic schedule is below the standard accepted dose, but some data [15] would suggest that lower doses of CAPE could have a more favourable therapeutic index in metastatic breast cancer because of a less incidence of suspension or delay.

The study by Saridaki et al. [9] described the results of a Phase I trial of the all-oral combination of metronomic VRB (starting dose 30) and CAPE (starting dose 800 mg/mq, bid on days 1–14 every 21 days). Thirty-six women were enrolled at eight escalating dose levels. The dose-limiting-toxicity (DLT) level was reached at oral metronomic VRB 70 mg and CAPE 1250 mg/mq. DLTs were febrile neutropenia Grades 3 and 4, diarrhoea Grade 4, and treatment delays due to unresolved neutropenia. The incidence of Grade 3 gastrointestinal adverse events was 16.5% (nausea/vomiting 11% and diarrhoea 5.5%), whereas Grade 4 diarrhoea was observed in 2.7% of the patients.

The administration of CAPE with a metronomic schedule could reduce the incidence of gastrointestinal disorders, increasing the compliance of the patients and assuring the best dose intensity of the drug. In our study, as well in the one by Dellapasqua et al. [7], the incidence of diarrhoea and nausea/vomiting was very low (4 and 16 events, resp.) with any reporting of Grade 3-4 events.

The major limit of the dose-finding part of the study was the low number of administered cycles. In order to assess the toxicity of prolonged treatment, 22 additional patients were treated with the recommended dose of 40 mg of VRB and CAPE 500 mg tid.

The low incidence of haematological toxicity observed in the dose-finding part of the study was confirmed.

In conclusion, the MTD of oral metronomic VRB was 40 mg/tot on days 1–3–5 of a week and it is the recommended dose for the ongoing Phase II trial.

The all-oral combination of VRB 40 mg thrice a week and CAPE 500 mg tid continuously was feasible and well tolerated also during prolonged treatment.

## Figures and Tables

**Table 1:** Patients' characteristics, Phases I-II.

*N* = 34		
Median age	72.5 (47–84)	
Median DFI (months)	82 (0–120)	

	*N*	%

Performance status (ECOG)		
0–1	26	76.5
2	8	23.5
Stage at enrolment		
Locally advanced	0	
Metastatic	34	100
Histology		
Ductal carcinoma	28	82.5
Lobular carcinoma	5	14.7
Other	1	2.9
Hormone receptor status		
ER+/PgR+	25	73.5
ER+/PgR−	5	14.7
ER−/PgR−	4	11.7
HER2 status		
HER2−ve	28	82.3
HER2+ve	3	8.8
HER2 unknown	3	8.8
Number of metastatic site		
1	7	20.6
≥2	27	79.4
Prior adjuvant therapy		
None	10	29.4
Chemotherapy	7	20.6
Endocrine therapy	7	20.6
Both	10	29.4
Prior therapy for metastatic disease		
None	5	14.7
Chemotherapy	2	5.8
Endocrine therapy	8	23.5
Both	18	52.9
Chemotherapy + trastuzumab	1	2.9
Number of previous chemotherapy treatments		
0	13	38.2
1	13	38.2
≥2	8	23.5
Prior treatments		
Anthracyclines	23	67.6
Taxanes	22	64.7

**Table:** (a)

	Level I VRB = 20 mg		Level II VRB 30 mg		Level III VRB 40 mg		Total	
	Per Pts	Per cycle	Per pts	Per cycle	Per pts	Per cycle	Per pts	Per cycle
Events G1								
Abdominal pain	0	0	2	3	5	15	7	18
Nausea	1	1	2	4	1	6	4	11
Gastric pain	0	0	1	1	3	3	4	4
Diarrhea	0	0	0	0	2	4	2	4
Vomiting	0	0	0	0	1	1	1	1
Stomatitis	0	0	1	1	1	1	2	2
Asthenia	0	0	1	1	2	2	3	3
Dyspnea	1	1	0	0	1	1	2	2
Dysgeusia	0	0	0	0	1	1	1	1
Anemia	0	0	0	0	0	2	2	2
Leukopenia	0	0	0	0	0	2	2	2
Increase in transaminases	0	0	1	1	0	0	2	1
Total	**2**	**2**	**8**	**11**	**17**	**38**	**32**	**51**
Events G2								
Abdominal pain	0	0	0	0	1	1	1	1
Nausea	0	0	1	2	2	2	3	4
Asthenia	0	0	1	2	3	4	4	6
Constipation	2	2	0	0	0	0	2	2
Total	**2**	**2**	**2**	**4**	**6**	**7**	**10**	**13**

**Table:** (b)

	Per pts	Per cycle
Events G1		
Abdominal pain	3	14
Nausea-vomiting	8	20
Gastric pain	4	4
Diarrhea	5	8
Stomatitis	2	2
Asthenia	4	15
Anemia	3	4
Neutropenia		
Thrombocytopenia		
Leukopenia	4	11
Transaminitis		
Dyspnoea	1	1
Neuropathy	3	4
Hand-foot syndrome	1	1
Nail changes	1	1
Muscular pain	3	5
Total	**40**	**90**
Events G2		
Abdominal pain	4	4
Nausea-vomiting	4*	4
Diarrhea		1
Stomatitis	1*	1
Asthenia	8	10
Dysgeusia	1	1
Neutropenia		1
Anemia	1	3
Hand-foot syndrome	1	2
Nail changes	2	2
Total	**22**	**29**
Events G3		
Neutropenia	1**	2
Thrombocytopenia	1	1
Leukopenia		
Neuropathy	1***	2
Hand-foot syndrome	1****	1
Gamma GT increase	1	1
Total	**5**	**7**
Events G4^∧^		
Neutropenia	1	1
Febrile neutropenia	1	1
Leukopenia	2	2
Total	**4**	**4**

*In 1 patient vomiting and stomatitis Grade 2 determined a dose reduction of VRB at 20 mg thrice a week.

**In 1 patient Grade 3 neutropenia was associated to Grade 1 Leukopenia; no dose reduction was required.

***Grade 3 neuropathy determined a dose reduction of VRB at 20 mg thrice a week.

****Grade 3 hand-foot syndrome determined a dose reduction of CAPE at 500 mg twice a day, until complete recovery to Grade 1.

^∧^See details in the text.

**Table 3:** Best response of the metronomic combination of VRB and CAPE according to RECIST criteria. *N* = 31.

	*N*	%
CR + PR	5	16.1
SD	9	29.0
Clinical benefit	18	58.1
